# A Perspective Study of Koumiss Microbiome by Metagenomics Analysis Based on Single-Cell Amplification Technique

**DOI:** 10.3389/fmicb.2017.00165

**Published:** 2017-02-07

**Authors:** Guoqiang Yao, Jie Yu, Qiangchuan Hou, Wenyan Hui, Wenjun Liu, Lai-Yu Kwok, Bilige Menghe, Tiansong Sun, Heping Zhang, Wenyi Zhang

**Affiliations:** Key Laboratory of Dairy Biotechnology and Engineering, Ministry of Education, Inner Mongolia Agricultural UniversityHohhot, China

**Keywords:** koumiss, metagenomics, single-cell amplification, bacterial diversity, low-abundant taxa

## Abstract

Koumiss is a traditional fermented dairy product and a good source for isolating novel bacteria with biotechnology potential. In the present study, we applied the single-cell amplification technique in the metagenomics analysis of koumiss. This approach aimed at detecting the low-abundant bacteria in the koumiss. Briefly, each sample was first serially diluted until reaching the level of approximately 100 cells. Then, three diluted bacterial suspensions were randomly picked for further study. By analyzing 30 diluted koumiss suspensions, a total of 24 bacterial species were identified. In addition to the previously reported koumiss-associated species, such as *Lactobacillus* (*L*.) *helveticus. Lactococcus lactis. L. buchneri, L. kefiranofaciens*, and *Acetobacter pasteurianus*, we successfully detected three low-abundant taxa in the samples, namely *L. otakiensis. Streptococcus macedonicus*, and *Ruminococcus torques*. The functional koumiss metagenomes carried putative genes that relate to lactose metabolism and synthesis of typical flavor compounds. Our study would encourage the use of modern metagenomics to discover novel species of bacteria that could be useful in food industries.

## Introduction

Koumiss, also named chige, chigo, arrag, or airag, in the Mongolian language, is a type of traditional fermented dairy product. It has been a popular food in Mongolia and Inner Mongolia of China for centuries (Zhang and Zhang, 2011). People in these regions used to consume koumiss during grand festivities and sacrificial offerings (Zhang and Zhang, 2011). The earliest record of koumiss production can be traced back to the Han Dynasty (BC202-AD202). This product had attained widespread popularity during the Yuan Dynasty (AD1271-AD1368) (Zhang et al., 2010b). Nowadays, koumiss is a common food for the local people of Mongolia and Inner Mongolia, although only in few of these areas, it is manufactured in an industrial scale. Koumiss does not only provide rich nutrients, including high contents of essential amino acids and vitamins, but is also believed to relieve a wide range of medical conditions and is beneficial for postoperative care (Jagielski, 1877; Thompson, 1879).

Traditionally, koumiss is commonly produced in wooden casks, containers made of animal skin or urns. Fermentation occurs naturally at ambient temperature after the addition of filtrated mare milk into the container with old koumiss, which serves as the starter culture (Zhang and Zhang, 2011). Koumiss is a good source of novel bacteria of biotechnology potential (Zhang et al., 2010a; Pan et al., 2011). Therefore, it is of intense interest to explore and preserve as many fermentation-associated koumiss bacteria as possible. During the last decades, a number of studies were performed to investigate the koumiss bacterial community (Wu et al., 2009; Hao et al., 2010), mainly studied by culture-, molecular biology- and pyrosequencing-based methods (Sun et al., 2010). Among these different approaches, the pyrosequencing-based method has provided the most comprehensive microbiota profile of koumiss independent from phenotypic traits and problems of cultivability of the individual microbes. However, the spectrum of functional genes coded by the koumiss bacteria and their fermentative capacities remain poorly characterized, particularly for the rare microbial populations.

The present study used the single cell genomics technique to analyze the bacterial metagenomes of 10 koumiss samples collected from Mongolia and Inner Mongolia of China. The current work has applied state-of-art technologies in investigating the bacterial diversity in dairy products. Our work has demonstrated the feasibility of discovering low-abundant taxa by applying the single cell metagenomics approach. The encouraging results would promote the development and application of novel approaches in tackling problems in a traditional field of research.

## Materials and Methods

### Preparation of Samples

A total of 10 koumiss samples were collected from Mongolia (MG14, MG15, MG16, MG17, and MG18) and Inner Mongolia of China (NM17, NM18, NM19, NM20, and NM21) for the metagenomics study. Samples were collected aseptically and were transported in dry ice.

One milliliter of each sample was pretreated according to the methodology described in Ward et al. (2013) with some modifications. Briefly, the samples were thawed in an ice bath for 3–5 min. After the samples melted, they were subject to low speed centrifugation to remove impurities and eukaryotic cell clumps. Prokaryotic cells were then pelleted from the milk sera by centrifugation at 13,000 × *g* for 15 min. The pellets were re-suspended in 2 mL phosphate buffered saline (PBS) with 1% Triton X-100 and incubated for 2 h at 37°C to lyse any remaining eukaryotic cells. Subsequently, bacteria were pelleted by centrifugation at 13,000 × *g* for 15 min and the pellets were re-suspended in 500 μL PBS. Finally, the centrifugation step was repeated once more to wash the bacterial cells.

### Gradient Dilution and Multiple Displacement Amplification

To detect the low-abundant bacteria, the bacterial suspension derived from each koumiss sample was serially diluted for subsequent amplification reaction. The cell number in each sample was roughly estimated under a microscope (Nikon, Tokyo, Japan) using a cell counting chamber (Qiujing, Shanghai, China). The dilution step was continued until the cell number in each bacterial suspension reached approximately 100. Multiple displacement amplification of the diluted cells was performed using the REPLI-g Single Cell Kit (Qiagen, Germantown, MD, USA) according to the manufacturer’s instructions.

### Library Construction and Sequencing

Amplified DNA was sheared randomly, and the fragments of approximately 500 bp were selected. After the library construction, PerkinElmer LabChip^®^ GX Touch and StepOnePlus^TM^ Real-Time PCR System were used for library quality inspection. Finally, 125-bp paired-end reads were sequenced on the Illumina HiSeq 2500 platform according to the manufacturer’s instructions.

### Data Analysis

#### Sequence Quality Check and Filtering

Raw reads generated by the sequencer might contain artificial reads of adapter contamination during the library construction. Therefore, three steps were performed to obtain a high-quality clean read dataset: (1) elimination of reads caused by adapter contamination; (2) removal of reads with an average score below a phred score of Q30, which was considered as the lowest cutoff for a high-quality base; (3) removal of reads with a significant excess of “N” (≥5% of the read). The downstream analysis was based on the clean data. Moreover, the statistical base quality, based on Q30 and the GC content, were calculated.

Alignments against the host genome were carried out to remove the host-originated contaminant sequences. Any host-originated reads were discarded before further comparison with bacteria (or viruses) genome reference sequences. To obtain more accurate results, the Burrows–Wheeler aligner (BWA) (Version 0.97a) MEM model was used in the alignments (Li and Durbin, 2009).

#### Taxonomic Assignment and Diversity

The web software Metaphlan was used for taxonomic assignment to genus and species levels (Segata et al., 2012). To compare the diversity of species within and between samples, we analyzed the alpha- and beta-diversity by the R-related package.

#### Read Assembly, Gene Prediction, and Annotation

To obtain more comprehensive information, we assembled the sheared fragments into genome (contigs). However, due to the presence of multiple species, which is common in metagenomic samples, we improved the bioinformatics genome assembling method normally used for single species analysis by integrating SPAdes (Version 3.6.2), in-house scripts, and metagenomic databases (Zerbino and Birney, 2008; Nurk et al., 2013).

The MetaGeneMark software was used for gene prediction of the assembled contigs (Noguchi et al., 2006). Redundant genes were removed using CD-HIT with the coverage of 90 and 95% identity (Li and Godzik, 2006; Fu et al., 2012). Relative abundances of the genes were determined by aligning high-quality sequencing reads to the gene catalog using the same procedure. The downstream discrepant analysis was based on the gene relative abundances. Gene annotation was performed by aligning the high-quality sequences against several public databases (namely NCBI non-redundant database, NR; Clusters of Orthologous Groups of proteins, COGs; Kyoto Encyclopedia of Genes and Genomes, KEGG) using BLAST (Altschul et al., 1997). A domain search was performed by using Interproscan (Mulder and Apweiler, 2008).

#### Nucleotide Sequence Accession Numbers

The sequence data reported in this study have been deposited in the SRA database (Accession No.SRP083102).

## Results

### Experimental Design and Sequencing

To detect the low-abundant bacteria in koumiss, the single-cell amplification technique was used to analyze the metagenomes of the samples. Three bacterial suspensions, each of approximately 100 cells, were derived from an independent koumiss sample by serial dilution. A total of 30 diluted suspensions were analyzed. Each diluted sample was given a different sample code, i.e., the sample identity number followed by 1, 2, or 3, representing the three separate dilutions. Based on a premise that some rare species will be present in one of the dilutions, multiple displacement amplification of the cells was carried out; and around 5 Gb data were generated for every koumiss bacterial suspension.

**FIGURE 1:**
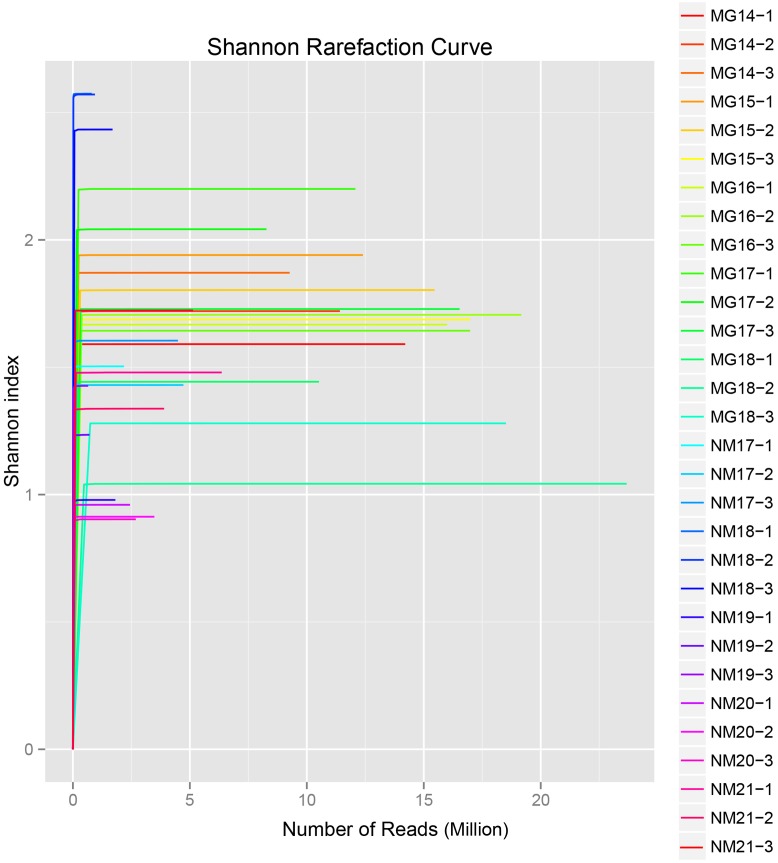
**Shannon rarefaction curves that estimate the microbial diversity of koumiss samples**.

**FIGURE 2:**
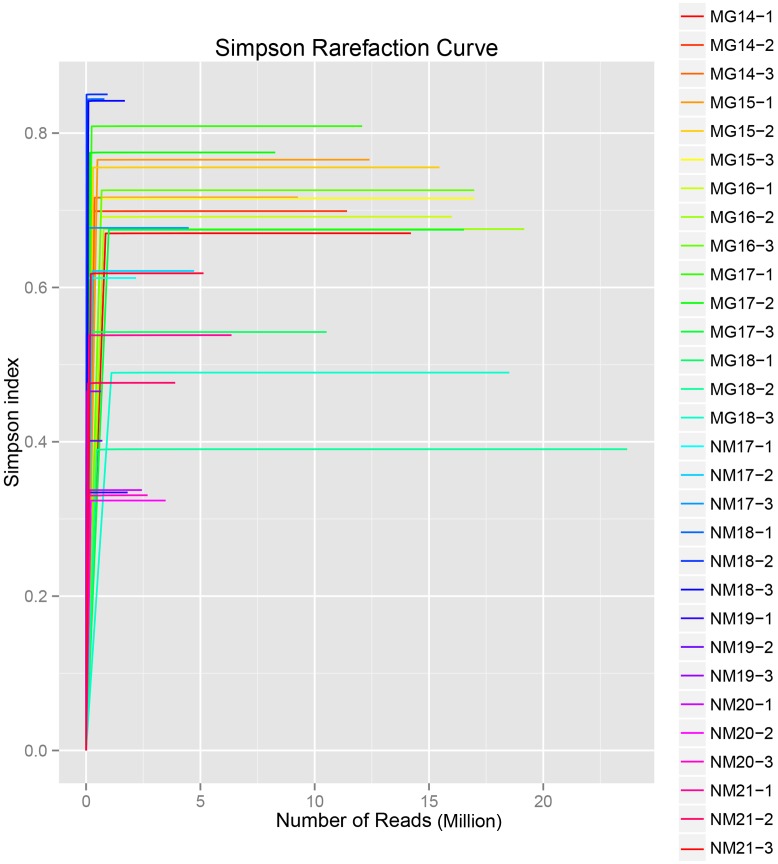
**Simpson rarefaction curves that estimate the microbial diversity of koumiss samples**.

**FIGURE 3:**
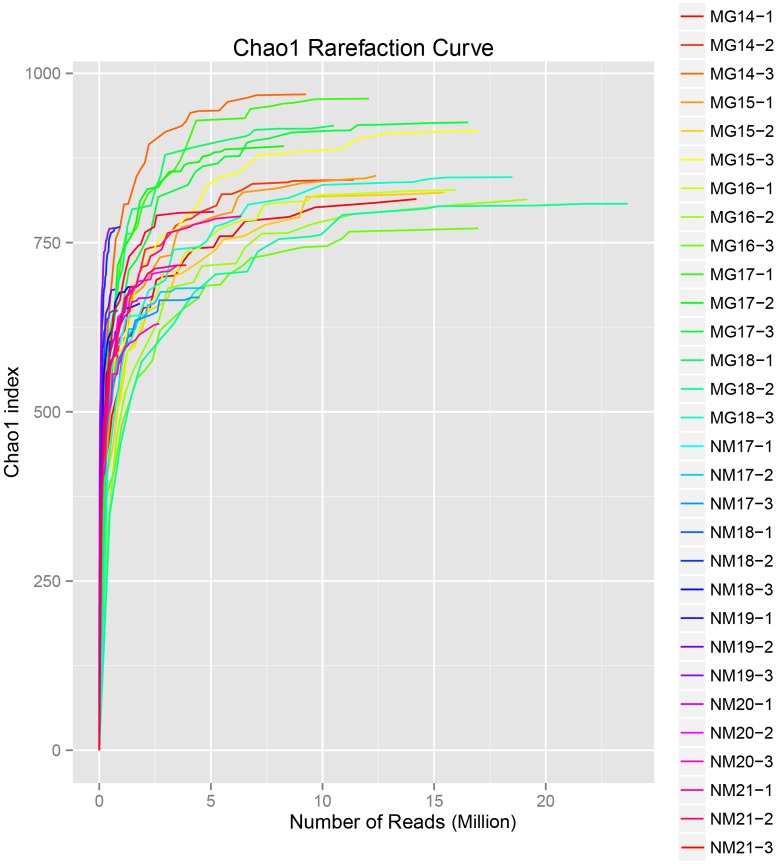
**Chao1 rarefaction curves that estimate the microbial diversity of koumiss samples**.

**FIGURE 4:**
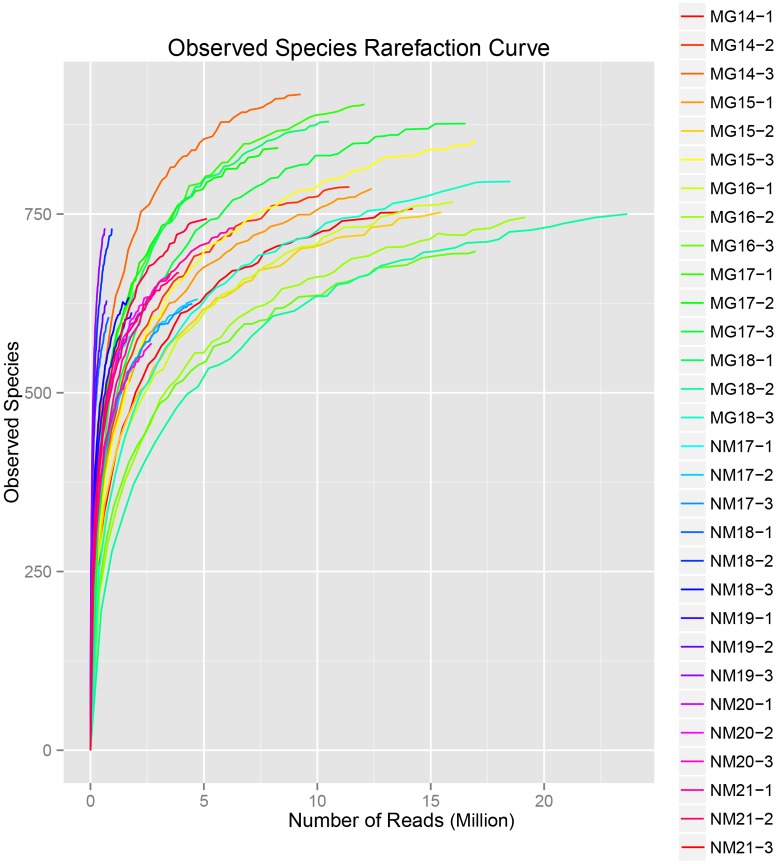
**Observed species rarefaction index that estimate the microbial diversity of koumiss samples**.

A total of 1,040,323,864 raw reads were generated from the 10 koumiss samples (a total of 30 bacterial suspensions). The average number of the reads for each 100-cell-suspension was 34,677,462 (**Supplementary Table S1**). After trimming and filtering of the unqualified sequences, we obtained 1,018,381,702 clean reads for all samples (**Supplementary Table S1**). The values of Shannon index, Simpson index, Chao1 index, and the number of observed species (**Figures 1**–**4**) showed that most koumiss samples had a high bacterial biodiversity. The Shannon–Wiener diversity curves showed that the sequence depth was adequate for all samples (**Figure 1**).

### Taxonomic Annotation

The high-quality sequences were assigned to different taxonomic levels to enable an in-depth analysis of the sample bacterial communities. With reference to some published studies on koumiss biodiversity (Wu et al., 2009; Hao et al., 2010; Sun et al., 2010), we classified the known and previously not reported koumiss-associated bacteria as common and rare taxa, respectively.

**FIGURE 5:**
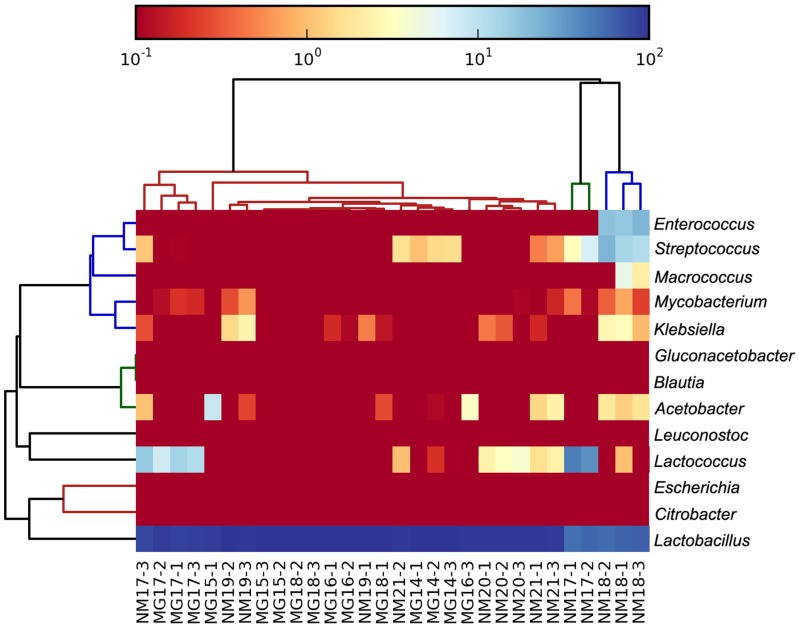
**Heatmap showing the relative abundance of bacteria detected in the koumiss samples at genus level**.

**FIGURE 6:**
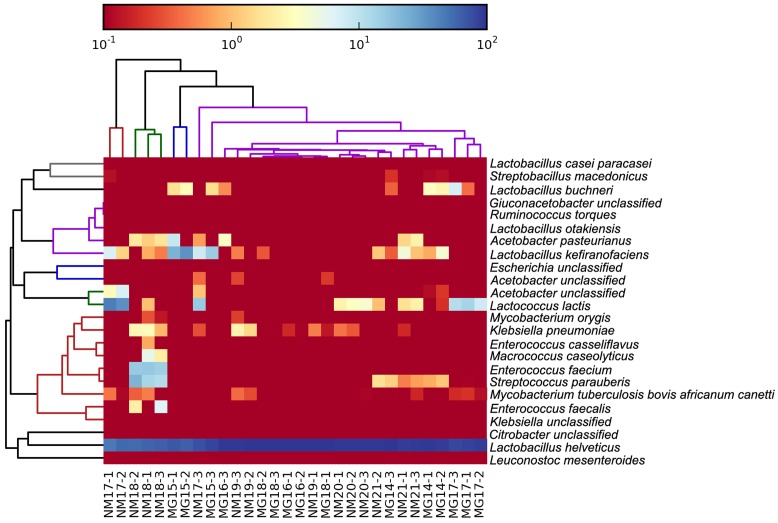
**Heatmap showing the relative abundance of bacteria detected in the koumiss samples at species level**.

The high-quality sequences represented 13 different genera (**Figure 5**). Three of them had an average relative abundance of over 1%, including *Lactobacillus* (*L*.), *Lactococcus*, and *Streptococcus*. Particularly, *Lactobacillus* and *Lactococcus* were the two most abundant genera found in the koumiss. The proportion of *Lactobacillus* in the samples ranged from 52.72 to 99.96%. Two members of this genus, *L. helveticus* and *L. kefiranofaciens*, were dominated among most koumiss samples (**Figure 6**). The species *L. buchneri* was present mostly in the Mongolian samples, while *Lactococcus lactis* was detected in most samples regardless of the sampling region.

### Metagenomic Assembly, Gene Prediction, and Functional Annotation

Assembling of the reads resulted in an assembly length of 614,392,623 bp. The N50 values for the assemblies ranged from 5,596 to 35,200 bp (**Supplementary Table S2**). The number of predicted genes in the koumiss samples ranged from 10,347 to 34,547, with an average length ranging from 647.82 to 985.33 bp (**Supplementary Table S3**). Although empirical gene functional analyses were beyond the scope of the current study, we annotated the koumiss bacterial microbiomes using the COG and KEGG databases, which predicted gene function largely based on sequence homology.

A total of 545 matches in the annotation output showed high homology to the lactose utilization genes (COGs category of carbohydrate and metabolism, G), and some of them were located within the same contig (**Table 1**).

**Table 1:** Lactose metabolism-related genes annotated in the koumiss bacterial metagenome.

Description	Gene name	EC numbers	Number of matches in the annotation output
Galactose-6-phosphate isomerase	*lac*A, *lac*B	EC:5.3.1.26	106
Tagatose 6-phosphate kinase	*lac*C	EC:2.7.1.144	12
Tagatose 1,6-diphosphate aldolase	*lac*D	EC:4.1.2.40	93
PTS system, lactose-specific IIA component	*lac*F (*lac*E)	EC:2.7.1.69	97
6-phospho-beta-galactosidase	*lac*G	E3.2.1.85	41
Beta-galactosidase	*lac*Z	EC:3.2.1.23	196


Some other sequences might code for putative genes within the COGs category of amino acid transport and metabolism (E), including sequences that corresponded to casein-degrading proteinases, the Opp system for taking up oligopeptides of 4–18 residues, and aminopeptidases (e.g., leucyl aminopeptidase, peptidyl-dipeptidase A, aminopeptidase N, proline iminopeptidase, and endopeptidase). Although some sequences shared high similarities with the aminotransferases specific for arginine, aspartate, methionine, and isoleucine, only one significant match was identified, which corresponded to a putative *S. macedonicus*-originated class I/class II domain (IPR004839)-containing aminotransferase specific for tyrosine and phenylalanine. Finally, a number of sequences shared high homology to the amino acid lyases, including S-ribosylhomocysteine lyase, argininosuccinate lyase, aspartate ammonia-lyase, cystathionine gamma-lyase, histidine ammonia-lyase, and O-acetylhomoserine (thiol)-lyase.

## Discussion

Koumiss is a popular traditional fermented dairy product in Mongolia and Inner Mongolia of China. Although a number of studies have been performed to investigate the bacterial diversity in koumiss, little information has been obtained regarding the genetic capacity of the koumiss microbiota. Here, we applied the single-cell amplification technique to analyze the koumiss bacterial metagenomes, particularly focusing on the low-abundant bacterial population.

The typical metagenomics approach has previously been applied to describe the koumiss microbiota. However, due to the high cost of achieving a deep sequence, the rare microbiota population in the samples is often covered inadequately. Thus, both the phylogenic and functional metagenomes of the minority bacteria remain limited. Our single-cell amplification method involved the serial dilution of samples to 100-cell suspensions. Owing to the low number of cells present in the diluted koumiss samples, only a limited amount of DNA would be extracted. The amplification step increased the quantity of DNA materials to be analyzed and hence facilitated the metagenome analysis of samples containing minute DNA amounts. One drawback of this method was the difficulty in ensuring that sequences of each taxon would be equally amplified in the process; therefore, the results obtained here could only reflect the relative abundances of sequences but not absolute quantities of identified taxa or functional genes. Yet, this would have little effect on our analysis as the present study differs from other published works in focusing the rare bacterial population. Data generated by this work provide complementary information to the underrepresented population. We believe the present approach is suitable for future analysis of bacteria diversity for different types of ecological environments.

The koumiss bacterial microbiota is mainly consisted of lactic acid bacteria (LAB) and some acetic acid bacteria (Zhang and Zhang, 2011). As expected, our dataset contained mostly sequences representing the LAB and acetic acid bacteria. Sequences corresponding to *Lactobacillus helveticus* were dominating across all samples. Besides, sequences representing the species, *L. kefiranofaciens. L. buchneri. L. kefiranofaciens. Enterococcus* (*E*.) *casseliflavus. E. faecalis. E. faecium. Leuconostoc mesenteroides. Lactococcus lactis*, and *Acetobacter pasteurianus*, were also found. The identification of sequences of different taxa may not be enough to show the viability of the bacteria, it nevertheless reflects the bacterial community at some point of the fermentation process. Miyamoto et al. (2010) suggests that the bacterial prevalence in the final fermented products is related to their acid stress tolerance. Generally, lactobacilli have a higher acid tolerance than lactococci, which may explain the high relative abundance of lactobacilli sequences present in our dataset. On the other hand, the frequent occurrence of *L. helveticus* in koumiss coincided with the observation of the dominance of *L. helveticus* sequences.

In our dataset, some sequences corresponded to *L. otakiensis*, which is a rare species that has never been reported in koumiss or other dairy niches. The species was firstly described and isolated from the non-salted pickling solution used in producing sunki, a traditional Japanese pickle (Watanabe et al., 2009). It was discovered by amplified fragment length polymorphism profiling based on the *rec*A gene. Since then, this species has not been reported to be associated with other food-related niches. Thus, it is likely that this bacterium belongs to the autochthonous flora of pickles. However, we cannot exclude the possibility that it was not found simply due to the sensitivity of detection method employed. *Lactobacillus otakiensis* can produce d-branched-chain amino acids, such as d-leucine, d-allo-isoleucine and d-valine (Doi et al., 2013). It has potential to be used in improving production characteristics of certain fermented foods (Kato et al., 2011).

Our study also identified sequences representing the species *S. macedonicus*, which has never been reported as a koumiss-associated bacterium. Instead, it is a starter culture present in Greek sheep and goat cheeses (Georgalaki et al., 2000). Some members of this species are able to produce exopolysaccharides, bacteriocins (Vincent et al., 2001; Anastasiou et al., 2015), and gamma-aminobutyric acid (Franciosi et al., 2015). Even though this species is frequently isolated from fermented foods, the original niche of *S. macedonicus* has been controversial (Guarcello et al., 2016). Not until recently, Papadimitriou et al. (2015) identified an acquired plasmid, pSMA198, from *Lactococcus lactis*. The plasmid was likely transferred via an ancestral genetic exchange event within a dairy product environment, hinting to the dairy origin of *S. macedonicus* (Papadimitriou et al., 2015). Similar to *S. thermophilus. S. macedonicus* is closely related to the commensals and opportunistic pathogens of the *S. bovis*/*S. equinus* complex.

The identification of sequences representing the species *Ruminococcus torques* was unexpected, as this bacterium is usually found in the gut environment. It is a normal human gut microbe that can degrade mucin oligosaccharides by constitutive production of secretory glycosidases (Hoskins et al., 1985). Recent clinical evidence shows that the fecal abundance of this species is altered in children with autism spectrum disorder; however, its role in the disorder remains unclear (Wang et al., 2013).

Surprisingly, some of the sequences represented potential pathogens. For example, *Klebsiella pneumonia* is an opportunistic human pathogen that resides in around 40% of human and animal guts. *Mycobacterium orygis*, previously called the oryx bacillus, is a member of the *Mycobacterium tuberculosis* complex that may cause human tuberculosis (Dawson et al., 2012). These two species have been reported in raw milk but not koumiss. Therefore, their presence could ascribe to the contamination during conventional koumiss production, especially under non- or low-aseptic manipulation conditions.

Bacterial metabolism plays an important role in forming the koumiss characteristics and quality. Microbial-based processes such as lipolysis and proteolysis are required for synthesizing koumiss aromatic and flavor compounds (Gesudu et al., 2016). Consistently, the bacterial metagenome contained sequences that potentially code for lactose degradation and proteolytic systems. Unlike the relatively simple lactose catabolic pathways, the LAB proteolytic systems are made up of a diverse array of enzymes (Chen et al., 2014). Compared with other detected koumiss LAB, the dominant species *L. helveticus* is characterized with a high proteolytic activity (Zhang et al., 2015). Most LAB possess only one cell-envelope proteinase that initiates the milk casein hydrolysis, whereas *L. helveticus* contains at least two of these enzymes, namely *PrtH* and *PrtH2* (Zhao et al., 2011). Thus, the high proportions of sequences corresponding to the strong proteolytic species of *L. helveticus* and proteolysis-related genes may link to the relatively high contents of peptides and free amino acids in koumiss.

One difficulty in industrial scale koumiss production is the control of flavor perception, as koumiss has been traditionally made by natural fermentation. Thus, it has been hard to define the flavor and the profile of key flavor components, particularly in the presence of the natural contaminants. The production of key flavor components is a result of fermentative and enzymatic degradation of amino acids, such as the branched-chain amino acids, methionine and aromatic amino acids (Ardo, 2006). Examples of flavor components include aldehydes, organic acids and esters, which are formed by transaminase (AT)-pathway (Helinck et al., 2004). This pathway is initiated by a transaminase that catalyzes the conversion of an amino acid to its corresponding α-keto acid (Helinck et al., 2004). Some sequences in our dataset corresponded to putative branched-chain and aromatic amino acid aminotransferases. Particularly, we found a putative aromatic amino acid aminotransferase I in the contig of the koumiss-associated species, *S. macedonicus*. Since our current work only annotated the microbiome *in silico*, our data are not enough to show that these identified gene sequences were indeed functional to produce the aforementioned koumiss flavor compounds. The presence of these genes nevertheless suggest that they are some possible candidates for such fermentative activities; yet, further experimental work would be required to elucidate their exact functional roles.

Moreover, we found sequences corresponding to other amino acid conversion pathways including amino acid lyases and threonine aldolase. The former enzyme catalyzes the conversion of methionine to methanethiol (Irmler et al., 2008), thus resulting in dimethyl disulfide and dimethyl trisulfide formation (Fernandez et al., 2000), while the latter one catalyzes the conversion of threonine to glycine and acetaldehyde (Ott et al., 2000). Similarly, merely locating these sequences within the koumiss microbiome cannot serve as definite evidence in their actual biological roles; future experimental confirmation will be required.

## Conclusion

The present study used a modified metagenomics method to analyze the bacterial microbiome of koumiss samples collected from Mongolia and Inner Mongolia of China. We characterized both the phylogenic and functional metagenomes of the rare species in koumiss; and our dataset reflects traits that are of biotechnology interest and potential. Our study has demonstrated for the first time the feasibility of incorporating the single-cell amplification techniques in detecting koumiss bacterial microbiota, as well as microbial contaminants. The techniques developed herein can be used in future studies to monitor changes in the koumiss microbiome along the fermentation process, with focus on the minority microbial population. Furthermore, other omics techniques, such as transcriptomics, metabolomics, can be coupled to the current metagenomics analysis in order to confirm the functions and metabolic capacity of the koumiss microbiome.

Technically, the next step of this work would be to optimize the current method. For example, by increasing the sample dilution before DNA amplification, the chance of uncovering rare and novel bacteria may be improved. On the other hand, an alternative sequencing technique that can generate long reads, such as Pacific Biosciences single molecule, real-time sequencing technology, can be employed to improve the genome assembling process.

## Author Contributions

WZ and HZ designed the study. WZ, GY, JY, and L-YK wrote the manuscript. QH, WH, WL, BM, and TS performed experiments. WZ and QH analyzed data. All authors reviewed the manuscript.

## Conflict of Interest Statement

The authors declare that the research was conducted in the absence of any commercial or financial relationships that could be construed as a potential conflict of interest.
