# Supplementary material for: A Perspective Study of Koumiss Microbiome by Metagenomics Analysis Based on Single-Cell Amplification Technique
Source: Front Microbiol. 2017 Feb 7;8:165. doi: 10.3389/fmicb.2017.00165 (PMC5293792; doi:10.3389/fmicb.2017.00165)
Supplement: Supplementary file 1 [file Table_1.DOCX]

**Table S1** Statistics from filtering process

| Sample | Raw Reads Number | Clean Reads Number | Clean Reads Rate(%) |
| --- | --- | --- | --- |
| MG14-1 | 33,980,432 | 32,811,800 | 96.56 |
| MG14-2 | 33,993,718 | 32,890,144 | 96.75 |
| MG14-3 | 34,227,904 | 33,468,690 | 97.78 |
| MG15-1 | 33,379,998 | 32,211,172 | 96.5 |
| MG15-2 | 33,755,188 | 32,777,326 | 97.1 |
| MG15-3 | 34,645,296 | 33,938,852 | 97.96 |
| MG16-1 | 34,107,900 | 33,530,732 | 98.31 |
| MG16-2 | 35,600,656 | 34,931,990 | 98.12 |
| MG16-3 | 35,269,380 | 33,966,982 | 96.31 |
| MG17-1 | 35,421,762 | 34,731,376 | 98.05 |
| MG17-2 | 34,598,030 | 33,983,222 | 98.22 |
| MG17-3 | 34,854,224 | 34,148,370 | 97.97 |
| MG18-1 | 35,455,360 | 34,905,200 | 98.45 |
| MG18-2 | 34,595,684 | 33,983,790 | 98.23 |
| MG18-3 | 33,912,050 | 33,332,430 | 98.29 |
| NM17-1 | 34,301,122 | 33,443,924 | 97.5 |
| NM17-2 | 34,254,352 | 33,655,402 | 98.25 |
| NM17-3 | 35,332,156 | 34,675,292 | 98.14 |
| NM18-1 | 35,402,364 | 34,764,894 | 98.2 |
| NM18-2 | 34,578,396 | 33,956,558 | 98.2 |
| NM18-3 | 35,223,942 | 34,603,906 | 98.24 |
| NM19-1 | 33,525,472 | 32,901,674 | 98.14 |
| NM19-2 | 34,125,108 | 33,497,870 | 98.16 |
| NM19-3 | 34,588,694 | 33,958,704 | 98.18 |
| NM20-1 | 35,017,996 | 34,431,982 | 98.33 |
| NM20-2 | 35,466,448 | 34,840,718 | 98.24 |
| NM20-3 | 34,461,114 | 33,755,846 | 97.95 |
| NM21-1 | 35,631,988 | 34,923,720 | 98.01 |
| NM21-2 | 35,313,632 | 34,668,928 | 98.17 |
| NM21-3 | 35,303,498 | 34,690,208 | 98.26 |
