# Supplementary material for: A Perspective Study of Koumiss Microbiome by Metagenomics Analysis Based on Single-Cell Amplification Technique
Source: Front Microbiol. 2017 Feb 7;8:165. doi: 10.3389/fmicb.2017.00165 (PMC5293792; doi:10.3389/fmicb.2017.00165)
Supplement: Supplementary file 2 [file Table_2.DOCX]

**Table S2**. Statistics of the assemblies

| Sample | Total length(bp) | N50 Length(bp) | N90 Length(bp) | Max Length(bp) | Min Length(bp) | Sequence GC(%) |
| --- | --- | --- | --- | --- | --- | --- |
| MG14-1 | 32,469,435 | 12,593 | 1,225 | 174,903 | 500 | 37.00% |
| MG14-2 | 33,701,377 | 12,494 | 1,275 | 173,458 | 500 | 37.95% |
| MG14-3 | 35,567,469 | 18,377 | 1,326 | 271,223 | 500 | 36.92% |
| MG15-1 | 16,050,386 | 14,268 | 1,747 | 182,968 | 500 | 40.56% |
| MG15-2 | 15,154,251 | 18,804 | 1,996 | 213,140 | 500 | 39.29% |
| MG15-3 | 14,846,496 | 17,612 | 1,506 | 183,047 | 500 | 39.20% |
| MG16-1 | 7,966,540 | 8,553 | 940 | 99,384 | 500 | 37.89% |
| MG16-2 | 8,140,859 | 7,657 | 826 | 139,588 | 500 | 37.85% |
| MG16-3 | 8,520,698 | 6,348 | 930 | 92,520 | 500 | 40.24% |
| MG17-1 | 22,660,653 | 8,767 | 1,118 | 157,714 | 500 | 38.95% |
| MG17-2 | 23,926,103 | 9,407 | 1,145 | 104,071 | 500 | 36.74% |
| MG17-3 | 17,519,291 | 7,743 | 1,033 | 144,441 | 500 | 38.35% |
| MG18-1 | 24,155,695 | 8,501 | 937 | 157,838 | 500 | 40.09% |
| MG18-2 | 17,724,496 | 5,596 | 887 | 87,387 | 500 | 39.27% |
| MG18-3 | 18,415,581 | 11,325 | 1,156 | 161,082 | 500 | 37.21% |
| NM17-1 | 21,780,847 | 26,230 | 1,784 | 220,628 | 500 | 38.63% |
| NM17-2 | 23,534,975 | 25,502 | 2,077 | 210,395 | 500 | 38.51% |
| NM17-3 | 23,325,335 | 23,573 | 1,990 | 257,610 | 500 | 38.94% |
| NM18-1 | 18,949,552 | 26,328 | 1,622 | 224,696 | 500 | 39.29% |
| NM18-2 | 19,982,901 | 22,743 | 1,565 | 272,422 | 500 | 39.26% |
| NM18-3 | 21,476,803 | 23,431 | 1,783 | 274,981 | 500 | 39.45% |
| NM19-1 | 16,298,694 | 31,498 | 3,971 | 260,266 | 500 | 39.20% |
| NM19-2 | 17,785,371 | 27,502 | 2,776 | 235,295 | 500 | 38.70% |
| NM19-3 | 15,534,706 | 35,200 | 3,522 | 208,156 | 500 | 39.65% |
| NM20-1 | 17,679,900 | 29,286 | 2,431 | 308,615 | 500 | 38.99% |
| NM20-2 | 24,851,843 | 25,334 | 1,920 | 311,378 | 500 | 37.21% |
| NM20-3 | 18,152,319 | 32,017 | 2,644 | 332,839 | 500 | 38.82% |
| NM21-1 | 28,014,600 | 17,947 | 1,391 | 308,445 | 500 | 40.51% |
| NM21-2 | 23,501,373 | 22,966 | 1,652 | 224,818 | 500 | 39.29% |
| NM21-3 | 26,704,074 | 22,101 | 1,619 | 307,819 | 500 | 40.15% |
