# Supplementary material for: A Perspective Study of Koumiss Microbiome by Metagenomics Analysis Based on Single-Cell Amplification Technique
Source: Front Microbiol. 2017 Feb 7;8:165. doi: 10.3389/fmicb.2017.00165 (PMC5293792; doi:10.3389/fmicb.2017.00165)
Supplement: Supplementary file 3 [file Table_3.DOCX]

**Table S3** Statistics of the predicted genes in different koumiss samples

| Sample | Gene Number | Gene Length(bp) | Gene Length/Genome(%) | Gene Average Length(bp) | GC Content in Gene Region(%) |
| --- | --- | --- | --- | --- | --- |
| MG14-1 | 32,841 | 25,250,112 | 77.77% | 768.86 | 38.13% |
| MG14-2 | 34,547 | 26,301,048 | 78.04% | 761.31 | 39.16% |
| MG14-3 | 33,108 | 27,194,205 | 76.46% | 821.38 | 38.11% |
| MG15-1 | 17,723 | 13,451,817 | 83.81% | 759 | 41.43% |
| MG15-2 | 17,005 | 12,917,541 | 85.24% | 759.63 | 40.09% |
| MG15-3 | 16,911 | 12,489,873 | 84.13% | 738.57 | 39.98% |
| MG16-1 | 10,347 | 6,702,981 | 84.14% | 647.82 | 38.59% |
| MG16-2 | 10,590 | 6,767,217 | 83.13% | 639.02 | 38.52% |
| MG16-3 | 10,921 | 7,017,921 | 82.36% | 642.61 | 40.84% |
| MG17-1 | 26,389 | 18,481,050 | 81.56% | 700.33 | 39.81% |
| MG17-2 | 24,841 | 18,561,612 | 77.58% | 747.22 | 37.81% |
| MG17-3 | 22,334 | 14,690,088 | 83.85% | 657.75 | 39.19% |
| MG18-1 | 27,771 | 19,192,725 | 79.45% | 691.11 | 41.28% |
| MG18-2 | 20,839 | 14,105,589 | 79.58% | 676.88 | 40.22% |
| MG18-3 | 20,300 | 14,649,474 | 79.55% | 721.65 | 38.37% |
| NM17-1 | 18,254 | 15,801,678 | 72.55% | 865.66 | 40.12% |
| NM17-2 | 20,301 | 17,311,602 | 73.56% | 852.75 | 39.93% |
| NM17-3 | 20,033 | 17,225,001 | 73.85% | 859.83 | 40.43% |
| NM18-1 | 15,382 | 13,440,804 | 70.93% | 873.8 | 41.00% |
| NM18-2 | 16,711 | 14,373,396 | 71.93% | 860.12 | 40.92% |
| NM18-3 | 18,220 | 15,654,483 | 72.89% | 859.19 | 41.08% |
| NM19-1 | 11,250 | 11,127,729 | 68.27% | 989.13 | 41.22% |
| NM19-2 | 12,508 | 12,126,210 | 68.18% | 969.48 | 40.65% |
| NM19-3 | 10,705 | 10,547,907 | 67.90% | 985.33 | 41.77% |
| NM20-1 | 13,389 | 12,340,353 | 69.80% | 921.68 | 40.77% |
| NM20-2 | 18,554 | 17,458,041 | 70.25% | 940.93 | 38.79% |
| NM20-3 | 13,838 | 12,758,001 | 70.28% | 921.95 | 40.53% |
| NM21-1 | 26,332 | 21,311,988 | 76.07% | 809.36 | 42.06% |
| NM21-2 | 20,822 | 17,378,682 | 73.95% | 834.63 | 40.82% |
| NM21-3 | 24,377 | 20,157,468 | 75.48% | 826.91 | 41.69% |
